# A Critical Enquiry into Variability of Insecticidal Net Use in Cambodia: Implications for Assessing Appropriateness of Malaria Elimination Interventions

**DOI:** 10.4269/ajtmh.18-0730

**Published:** 2019-04-15

**Authors:** Charlotte Gryseels, Melanie Bannister-Tyrrell, Sambunny Uk, Srun Set, Sokha Suon, René Gerrets, Koen Peeters Grietens

**Affiliations:** 1 Institute of Tropical Medicine, Antwerp, Belgium;; 2 National Center for Parasitology Entomology and Malaria Control, Phnom Penh, Cambodia;; 3 Amsterdam Institute for Social Science Research, University of Amsterdam, Amsterdam, The Netherlands

## Abstract

Distributing long-lasting insecticidal nets (LLINs) to individuals living in malaria-endemic regions is a cornerstone of global malaria control. National malaria control programs aim to achieve “universal coverage” of at-risk populations to reach LLINs’ full potential to reduce malaria, progress of which is then measured by indicators constructed from standardized questionnaires. Through an exploration of variability in LLIN use in Cambodia, we argue that indicators of universal coverage of LLINs are not sufficiently commensurate with the realities they are intended to measure, limiting the suitability of the data to serve program and policy purposes in a malaria elimination era. Reflecting on the various sources of variability in LLIN use, we apply and extend the concept of “appropriateness” as a third prong to the widely used “efficacy” and “effectiveness” criteria for evaluating LLINs as a tool for malaria prevention. Describing first the different dimensions of the intervention and the sociocultural context separately, we will further show how the variability underlying both is affected and induced by inappropriate aspects of the intervention and the measurements of its impact. We consider the gap between “net use” and the numerical representations of such local net use justifies further exploration of potential strategies to improve LLIN use in subgroups where persisting malaria transmission clusters.

## INTRODUCTION

The distribution of long-lasting insecticidal nets (LLINs) to communities living in malaria-endemic countries is still a cornerstone of malaria control today. Because of a successful scaling-up of control measures, including LLINs and insecticide-treated nets (ITNs), and an overall declining malaria incidence, malaria elimination in many countries has once again been declared feasible since 2007. In the same year, the WHO began recommending that all malaria control programs ensure “universal coverage” of freely distributed or highly subsidized LLINs in populations at risk.^1^ Universal coverage is currently defined by two indicators^2^: 1) the percentage of people who have access to LLINs in the household and 2) the percentage of people reporting having slept under an LLIN the previous night. The WHO defines “operational success (as opposed to a target of 100% coverage) as the observation in surveys of at least 80% coverage in terms of these indicators.”^2^ To measure whether countries have achieved “operational success,” The Roll Back Malaria Partnership provides standardized household questionnaires for use in malaria indicator surveys. There are indicators for household ownership of (sufficient) LLINs and an indicator for individual LLIN use, operationalized as the respondent reporting to have slept under a LLIN the night before the survey.^3^

When assessing whether universal coverage with LLINs has been achieved and the extent to which ongoing malaria transmission is attributable to “residual” transmission,^4^ indicators of LLIN use are necessarily abstracted from the sociocultural context to allow comparison between settings and the generation of generalizable strategies for malaria control and elimination. However, variability, often at fine socio-geographical scales, exists in the extent to which communities and/or individuals “comply” to interventions such as LLINs. Long-lasting insecticidal nets are objects that can affect and are affected by sleeping arrangements, housing structure, the spatial organization within a house, and behavior from visiting friends and relatives.^5–9^ Acceptability studies across the malaria-endemic regions of the world have long since shown a discrepancy between the designs of LLINs and targeted users’ social requirements of LLINs and other bed nets.^6,9–14^ These important findings have seldom prompted the malaria community to revise LLIN programs or impacted on how the induced variability in LLIN use is measured through indicators of “ownership,” “coverage,” and “use” when evaluating the average effectiveness of LLIN distribution programs. The impact of numerous social, cultural, economic, and environmental factors^5,7,9,15,16^ is averaged at the level of the lowest administrative unit (e.g., district or country) required for methodological and reporting purposes. Although stabilizing variability in LLIN use across time and place renders it measurable (i.e., quantifiable), this reification also obscures the phenomenon that is being measured, which is the extent to which people are *effectively* protected from biting vectors while sleeping at night, in (all) places where they are exposed. Although gaps between simple indicators and their measurement targets are ubiquitous, in a malaria elimination context, failure to recognize and capture variability in LLIN use leads to assumptions about causes of ongoing malaria transmission once universal coverage is assumed, and accordingly, to the prioritization of specific types of public health interventions and research.

In addition to the indicators, the intervention itself is also at best minimally adapted to the diverse contexts in which LLINs are intended to be used. Failure to localize LLINs can be understood as relating to the (in)appropriateness of the intervention. Although this concept has been formally applied to clinical care,^17,18^ there has been minimal extension of the concept of appropriateness in global health, especially for preventive public health interventions, which are instead often assessed in terms of the efficacy–effectiveness paradigm. Unlike concepts such as effectiveness, which estimate average net intervention effect under real-life conditions, the concept of appropriateness was introduced in clinical care to expand the evidence base underlying clinical decisions about which procedures and tests are appropriate for patients in a certain setting or context, as well as to focus attention on reducing unwarranted variation in medical care practices.^17^ In this article, we aim to adapt and expand the concept of appropriateness to the context of malaria control and elimination interventions and go beyond “acceptability” studies. We define “appropriateness” as 1) the extent to which LLINs as an intervention (both their design and distribution) align with the conditions and associated requirements of the contexts in which they are intended to be used and 2) how well the indicators that aim to capture its effect align with the real-life variability in LLIN use.

## METHODS

### Study design.

Ethnographic research was embedded in a community-randomized trial investigating the impact of mass distribution of personal topical repellents in addition to the distribution of LLINs on malaria prevalence in Ratanakiri province, Cambodia, between 2012 and 2013. The ethnographic research was conducted ancillary to the trial. The social science field team worked independently of the trial team and developed relationships of trust with the study population during extended stays in the study villages. Variability in LLINs was an emerging theme during ethnographic fieldwork and in an additional structured observation survey of personal topical repellent and bed net use (including LLIN, ITN, and non-treated nets), we identified population subgroups who did not use LLINs or other bed nets, or used bed nets less frequently because they were using the provided repellent (for more details, see Ref. 19). After the conclusion of the trial, the social science team carried out an explanatory qualitative study in 2014, aiming to further characterize these contexts and conditions of variability in bed net use and nonuse, the results of which are presented in this article. This study made use of in-depth (*n* = 130) and informal interviews (*n* = 21) with both the identified subgroups described previously, as well as with other relevant key informants such as health center staff, village malaria workers (VMWs), village chiefs, and other community members.

### Study site.

Research was conducted in Ratanakiri Province, a remote region of northeastern Cambodia bordering Vietnam. Several indigenous populations inhabit this region, including Jarai, Tompuon, Kreung, Lon, Prov, Kavet, Kachok, and Lao peoples, as well as a rapidly growing Khmer migrant population, the predominant ethnic group in Cambodia. Most of our ethnographic investigations during the trial took place in two predominantly Jarai villages in Oyadao district, two Tompuon villages along the Tonle San River in Voen Sai district, and one Tompuon village located further south in the province near the Srepok River in Lumphat district. We conducted additional observations and informal conversations in the district town centers and the district health centers, and private practices in the district centers and in the provincial capital Banlung. After the end of the trial, the explanatory qualitative study was conducted in the villages selected for the structured observation survey, which included three Jarai villages in Oyadao district, three Kreung villages in Ochum district, three Tompuon villages in Lumphat district, and one Tompuon and one Kreung village in Voen Sai district.

Malaria is endemic at low prevalence in Ratanakiri, primarily caused by *Plasmodium vivax* and *Plasmodium falciparum*, but *Plasmodium malariae* and *Plasmodium ovale* infections also occur.^20^ As measured by passive case detection, overall annual incidence rates per 1,000 inhabitants of all *Plasmodium* species decreased from 85.9 in 2010 to 30.4 in 2014 in Ratanakiri.^21^

The Cambodian National Malaria (CNM) Control Program implements the WHO-recommended malaria control strategies through provincial health departments, operational health districts, local health centers at district and commune levels, and at community level through VMWs, who are trained to diagnose and treat malaria with rapid diagnostic tests and first-line artemisinin-combination therapies.^22^ In collaboration with the health centers, VMWs also manage the distribution of LLINs, one for every person registered in a malaria-endemic village. The national malaria control guidelines stipulate this distribution should occur every 2 years. Beyond these official control measures, villagers can obtain non-treated bed nets (“market nets”), non-treated hammock nets, personal topical repellents, and mosquito coils at local markets, sometimes at prices subsidized by international non-governmental organizations. A range of unlicensed therapeutics, including pharmaceutical drug “cocktails” and traditional remedies, are readily accessible in the pluralistic health-care system.

### Data collection.

Ethnographic research was carried out using interviewing and participant observation as the main data collection techniques. All interviews were either carried out in English and translated to Khmer or were conducted in Khmer. When informants did not speak Khmer fluently, village guides or other key informants of the same village assisted in conducting interviews and were appropriately reimbursed for their time.

Informal conversations with people in the villages and at the health centers during our stays in the villages—during the trial, four visits of 2 months each, and, for the explanatory qualitative study, one visit of 2 months—constituted an important part of our data collection process. Participant observation was used as an ethnographic technique that enabled the researchers to contrast people’s actual behavior to people’s reported behavior. It included both continuous informal conversations and observations of people’s practices in their homes, in village, and farm settings, and during social or ceremonial events taking place in the village. Both informal conversations and observations were transcribed in field notes, and each single observation and conversation constituted a separate data entry in the final database.

### Sampling.

Participants for in-depth and informal interviewing were theoretically selected and gradually included from the village populations (including self-reported users and nonusers of both LLIN and non-treated market nets), public health center staff, private health-care providers, and provincial health department staff, usually through snowball sampling techniques. Other respondents were identified from a quantitative structured observation study (methods described in detail in Ref. 19) and included everyone from the following subgroups: household leaders of households 1) who were observed to not use any bed nets (*n* = 29) or 2) who self-reported using bed nets less frequently because of the availability of the trial repellents (*n* = 60). Informants were additionally classified according to variables, such as gender, age, subsistence strategies, locality, ethnicity, and occupation, to allow for internal variation and comparison.

### Data analysis.

Preliminary data were frequently reviewed and analyzed in the field, concurrent with data collection. Although we made use of question guides, interviews were nevertheless open to allow for emergent information. In the initial phase of research, raw data were coded inductively in Microsoft Word. When preliminary coding was performed, new hypotheses often emerged and the question guides were adapted to reflect the new hypotheses. These were further tested in the field until theoretical saturation was reached. A final coding tree was developed based on the results of the analytic process during fieldwork and applied to the data. NVivo 9 Qualitative Analysis software (QSR International, Doncaster, Victoria, Australia) was used for all final data management and deductive analysis. Coding queries were used to test relationships between codes or between codes and attributes of respondents.

### Ethical approvals.

The Institutional Review Board of the Institute of Tropical Medicine, Antwerp, and the Cambodian National Ethics Committee for Health Research granted ethical approval. All participants were explained the objectives of the study, the risks and benefits involved, the type of questions that were going to be asked, the use of the results for publications, and their right to stop the interview and withdraw participation at any time. They were then free to provide their oral consent to participate in the study. All interviewers followed the Code of Ethics of the American Anthropological Association.

## RESULTS

The concept of appropriateness is intricately tied to the variability in net use we introduced earlier. Defining first the different dimensions of the intervention and the sociocultural context separately, we will go on to show how the variability underlying both is affected and induced by inappropriate aspects of both the intervention and the measurements of its impact.

### The sociocultural context.

The indigenous ethnic groups of Ratanakiri originate from different geographic and linguistic lineages. Nevertheless, they are often closely related, and as neighbors, they interact and intermarry to the extent that it would be wrong to view each ethnic group as a separate entity from the larger social system and ecological niche they share.^23^ Most indigenous communities in Ratanakiri practice slash-and-burn agriculture, including cultivation of dry rice (“upland rice”) and various vegetables such as eggplant, gourd, corn, cucumber, sesame, and yam at farms cleared in the forest.^23^ Each village has its own forest territory, where mostly nuclear families work farm plots.^24^ Entire families frequently stay in bamboo houses constructed at their farms because of the distance to the village house, spiritual relations with the cleared lands, and the necessity to protect crops from wild animals and roaming cattle at night and during the day. Livelihood practices are therefore intimately interwoven with sleeping arrangements. Household composition at farm and village houses is fluid: teenagers often move between villages and fields for social and economic activities^25^; children sometimes remain in the villages with grandmothers to attend school when the Khmer teachers are present; and adult men come back for a night or two to participate in social events such as general drinking parties, funerals, weddings, or other ceremonies that are mostly characterized by drinking rice wine.

People frequently engage in forest activities such as hunting and logging, which primarily occur in the evening or nighttime, and overnight stays are common. Such activities are usually performed by groups of men in the associated forest spaces of a particular village. Other forest activities, such as collecting wild roots, vines, and fruits, are carried out by larger groups of men, women, and children during certain times of the year. Sleeping spaces are flexible and not reducible to a predictable rotation of mobile individuals in one sleeping space.

### The intervention.

There are three distinct dimensions of LLINs as an intervention: the product, procurement, and the distribution strategy for the product.

#### The product.

Long-lasting insecticidal nets are designed to reduce the incidence of malaria by offering personal protection to users from exposure to infectious biting vectors. This protection is assured by three distinct functions: 1) forming a protective physical barrier between the mosquito and its blood meal, 2) killing the mosquitoes that come into contact with the insecticide with which the nets are impregnated, and 3) the repellency effects of the insecticide that reduces mosquito entry into the house where LLIN users sleep. The insecticidal properties of nets also offer household-level and community-level protection by reducing the overall population of infected vectors, if used sufficiently at individual level. Implicitly, therefore, LLINs are intended for use indoors and/or in locations where they can be hung, when people are sleeping, at hours of the day (usually nighttime) that coincide with vector biting peaks. At the time of our fieldwork, Olyset nets (Olyset^®^ Net; Sumitomo Chemical UK PLC, London, United Kingdom) were distributed in Ratanakiri. The company presents its product as a quality LLIN because its main characteristics include the follows: 1) “hybrid polymer and controlled insecticide release technology to repel, kill, and prevent mosquitos from biting for up to 5 years”; 2) being “washable,” meaning they have “passed the > 20 washes test required by the WHO to be designated an LLIN”; and 3) being very strong because of their thick fibers (180 deniers).^26^ In addition, Olyset net mesh sizes are relatively large (4 mm) than many other brands of LLINs (usually 1.6 mm) to increase ventilation.^26^

#### The appropriateness of the product in the sociocultural context.

The appropriateness of the nets available in Ratanakiri (both Olyset LLIN and non-treated market nets) in relation to varying housing and environmental factors directed use: small farmhouses are usually only able to fit the smaller sized distributed LLIN and larger village houses are able to fit the larger market nets, which despite indicating variable LLIN use, may nonetheless be a suitable arrangement for malaria prevention as sleeping at farmhouses has been shown to increase malaria risk.^20^

Our informants considered the fabric of the LLINs distributed by the National Malaria Control Program at the time of this study simultaneously too hard and too fragile, and especially, the mesh sizes too large. Mesh size is an important characteristic of nets that induces the perception of protection and privacy: large mesh sizes are reported to let in “all sorts of insects,” including mosquitos, despite the insecticide; they form less of a protective barrier between the safe and domesticated “indoor” and the untamed “outdoor” when sleeping in farmhouses located next to forests that harbor wild animals and vindictive spirits; and they do not offer any privacy in homes where newlyweds are sleeping next to their parents. Contrary to the LLINs, untreated nets from local markets or traveling salesmen are soft and accommodate large families, and are perceived to have smaller mesh. Moreover, their mesh sizes are smaller than LLINs, which leads to the empirically sound perceptions of sufficient insect protection as well as greater privacy. This difference in acceptability between the distributed LLINs and the market nets leads to differences in actual use; those families that can afford them and have the relatively large space that is required to hang market nets are more likely to be using the market nets on a regular basis.

#### The procurement processes.

Variability in LLIN use can be related to priorities and decisions at national and international levels concerning procurement and mass distribution of standardized, quality-assured (i.e., WHO Pesticide Evaluation Scheme [WHOPES] approved) LLINs for implementation across the world’s highly diverse malaria-endemic regions. The malaria-epistemic community,^27–29^ which is global in outlook and with rapidly expanding membership in the modern malaria elimination era, is a product of current global health hegemonies that emphasize rapid up-scaling of interventions globally, innovation, and setting ambitious targets to drive global progress toward the ultimate goal of malaria eradication.^30^ Accordingly, malaria control programs are advised to establish single national policies for achieving universal LLIN coverage that ensure cost-effective and equitable provision of LLINs to all populations at risk of malaria. Long-lasting insecticidal net distributions are therefore preceded by clearly defined procurement criteria. First, the WHO provides recommendations to national malaria control programs for selecting approved LLINs, based on bioassay tests and net durability tests performed by the WHOPES. Second, based on these recommendations, donors such as the Global Fund construct a list of authorized suppliers of WHOPES-approved nets that national malaria control programs can direct donor funding toward. Procurement is envisioned as an open tender process to acquire contracts for the cheapest possible internationally standardized LLINs.

#### The appropriateness of the procurement in the sociocultural context.

Although procurement is envisioned as an open tender process, in Cambodia, it has been reported to involve corrupt bidding practices.^31^ As a consequence, donor control and financial auditing of the execution of international policies and programs have tightened, which appears to have had the effect of inducing delays in the implementation of LLIN distributions. Accordingly, there has been relatively little emphasis in these procurement processes toward selecting LLINs with characteristics that are most appropriate for the specific target populations.

#### The distribution.

The LLINs purchased at the national level then need to be distributed to populations at risk of malaria. There are several distribution strategies possible. Almost universally applied is the free-of-charge distribution at the local level of a predefined number or nets per person (usually one net per two household members). Additional strategies exist, such as socially promoted and subsidized nets for sale at markets; and hang-up keep-up strategies, to tackle the problem of new nets being saved for the future instead of as replacement of the current nets.^32–35^

Cambodian national malaria policy calls for distribution of LLINs to all people living in malaria-endemic regions. The program aims to ensure that the known barriers to the effective scaling-up of insecticidal nets are adequately addressed: 1) LLINS are distributed locally and free of charge, so there are no financial barriers; 2) LLINS are distributed one per one person per village unit, so every single household member should have access to an LLIN; and 3) distribution rounds occur once every 2 years, assuring continued access to effective LLINs.

#### The appropriateness of the distribution in the sociocultural context.

This also translates to distribution practices at district and village level, where several barriers emerge to assuring the LLIN distribution runs as envisioned by international donors. Local distribution rounds are organized by district health center staff, with the support of provincial health departments, in collaboration with village authorities such as village chiefs or VMWs. On the announced day of the LLIN distribution, registered residents must present their “bed net booklet”—that is, the administrative paperwork that registered residents receive to receive LLINs—to the health center staff and have it stamped at the central location in the village where the distribution is organized. In addition to the many Khmer migrants that are not registered in Ratanakiri because they have recently migrated from another province where they are registered, there are also registered indigenous inhabitants who do not receive the “bed net booklet” as they were away from the village center at the time the booklet was distributed. Difficult social relations may exist with key community members such as the village chief, VMW, or health center staff organizing the distribution rounds, which causes some villagers to be “omitted” from the village list. Key informants from villages and the health system also report that somewhere on the hierarchical line (from government official, provincial health department, and health center staff to village chief or VMW) LLINs are frequently withheld from distribution because of a system of social privilege in Cambodia that allows for and enables government officials to keep items such as LLINs for other family members or other purposes.^36^ Related distributional practices therefore also reflect and reproduce the social inequalities inherent in a system of patronage.^36^

### Variations in LLIN use in time and place.

Assumptions of universal coverage are further challenged by place-based and temporal variations in LLIN use.

#### Place-based variation.

Intended distribution of one LLIN per one person aims to cover individuals in one sleeping space and does not consider the varying sleeping spaces individuals use. When families are divided throughout several residences, it is not straightforward for the mobile household member to carry a LLIN from sleeping space to sleeping space and leaving other household members exposed. As there are varying needs in relation to the social events in the village or far away fields that need attending, and in relation to child care in the village in absence of most family members working at fields, there is always some proportion of the population requiring flexible and multiple sleeping spaces, and therefore less likely to be using a LLIN. Net priorities (for both LLIN and market nets) also varied and related to the multiple residence system; families in Ratanakiri often preferred to keep their market nets in the village house simply because the house was structurally better suited to contain the large-sized market nets needed to accommodate large families. Long-lasting insecticidal nets were often preferred for farmhouses because insect nuisance was perceived to be higher at farms and people reported not being able to sleep there without a net separating them from nuisance insects. Especially in such high nuisance settings, the mesh sizes were considered important and large mesh sizes were considered inappropriate, given the insects that were perceived to be able to enter the net.

#### Temporal variation.

Long-lasting insecticidal net use varies in accordance with agricultural calendars, with time since the last LLIN distribution, and over the life course. Indigenous men and women engage frequently in cultural events and ceremonies such as funerals, weddings, harvest, or planting ceremonies and other ceremonies to satisfy spirits, which occur both variably and at fixed times of the agricultural cycle. These events are always linked to the consumption of rice wine. Although now drinking may be more often linked to “parties,” these events lead to most of a village population or a particular social subgroup going to bed late at night and often too intoxicated to find, or care about finding, a bed net to sleep in. Such events are frequent in Ratanakiri, and therefore could skew standardized questions related to LLIN use that expect a straightforward answer. People who report to always sleep in a LLIN in surveys may in fact usually do so but not when they return home drunk from such (frequent) rice wine ceremonies or parties.

In relation to the temporal decline in net quality, both LLINs and market nets tend to tear quickly in local households in Ratanakiri.

Yes we liked to sleep in it [distributed LLIN] because it had insecticide to protect from mosquitoes. But my wife washed it often because my children always pee on the bed net. So it wore out of insecticide and became fragile. It has big holes so after it wore out of insecticide, the mosquitoes and other small insects could enter it. (Kachok farmer)

As nets are commonly urinated on by children and picked at by chickens and mice, frequent washing of nets is common as cleanliness is considered a social virtue. Although people are aware that frequent washing of LLINs reduces the effect of the insecticide and makes the nets tear more quickly, they nonetheless have to wash the nets weekly to avoid the smell becoming unbearable, which highlights the contrast between the required durability and stated durability of the distributed LLINs as exceeding (only) 20 washes. The wear and tear LLINs incur under these conditions led many of our informants to stop using the LLINs relatively quickly after distribution. The gap between projected LLIN durability and their actual lifespan is another example of temporal variability that cannot be measured with indicators that assume a temporally stable reality. Indeed, the most persistent comment was that the inherent low quality of the distributed LLINs was thought to make the nets tear “by themselves.” Some informants told us they perceive the low quality of LLINs to reflect the low social status the national malaria control program affords them as “ethnic minorities.” They did not think the LLINs lasted longer than a year after distribution, and therefore many experienced a gap in protection between distributions during which they simply did not have nets to use. Thus, if they report in surveys that they do not use an LLIN, this measure of “noncompliance” in part refers to a lack of access to a good and durable net for the local context. Although those who can will buy market nets to use in the time between distributions, most indigenous farmers cannot afford (enough) market nets to cover all household members.

Another temporal dimension of the variability in LLIN use relates to different age groups and life stages among indigenous peoples in Ratanakiri. Families will prioritize the protection of small children who sleep together with their mothers.

There are many mosquitoes until 8 to 9 pm. I worry about small children; I let them sleep in the bed net. As I am an adult, there is less of a problem. When my children get fever, they get convulsions. I spent 100,000 riels [∼ $25 USD] for treatment for my children at the health center. He had an infusion there for three days. (Tompuon farmer).

Children are perceived to be more vulnerable to illness and the biomedical treatments required to cure them are thought to be more expensive. However, in this setting, malaria risk clusters in adolescent and adult men, particularly when sleeping and working in or near the forest.^20,37–39^ Men and older male children were given least priority to sleep protected and revert to sleeping on mats with blankets or hammocks without nets when distributed LLINs are no longer fit for use. Men particularly go through different life stages that can be linked to different uses of bed nets. Groups of young adolescent boys sleep at friends’ houses, in empty houses, or in communal village houses with hammocks (mostly without nets), avoiding the fixed sleeping spaces that more readily enable use of LLINs within their family home(s). Likewise, the sociality enabled by hammocks is in line with the social events adolescent boys prefer to participate in, and the sleeping sites that are available to them during such social events are simply more hammock friendly.

Like me, my bed net has been hung in the village house, but I sometimes went to sleep at the farm with friends. For example, there is a man who has a big house, his house has enough spaces for boys to sleep together, around 16–17 boys sleeping with hanging hammocks, but some people prefer to sleep with bed net. (Tompuon teenage boy)

Social custom further affords adult bachelors the freedom to participate in various evening social activities and sleep in various locations as preferred; a practice also facilitated by lightweight and easy-to-carry hammocks. When they get married, men start sleeping together with their wives in a bed net, offering additional privacy in shared houses with extended families. However, when a man loses his wife, either because of untimely passing or to divorce, or remains a bachelor even in later years, it is common for him to stop using bed nets, stressing again the importance of privacy and the required size of the net and mesh that affords a sense of privacy when committing to consistent net use. Men who state to always sleep in a bed net when asked in a survey will likely only do so while they (still) require that privacy.

## DISCUSSION

Although data derived from malaria indicator and other surveys estimate a high level of bed net use in Ratanakiri and implicitly assume this use is continuous and consistent,^40,41^ our research demonstrates that there is considerable variability in local sleeping patterns and net use that resists being captured by quantitative indicators derived from questions that assume a stable pattern or straightforward response. In both policy and research circles, the default, or implicit, assumptions about the generic social context in which LLINs are used include that sleeping arrangements are stable, each net will be used by up to two adults, and that distributing sufficient LLINs based on household size to every household at-risk, coupled with behavioral change communication interventions, should achieve universal coverage. Although it has been recognized that the net use gap, referring to the difference in numbers between household ownership of at least one LLIN and household use of LLINs, is not simply due to a failure of behavioral change communication but to a lack of intra-household access,^42^ our results show that deriving “use” from the questions that construct these indicators further overlooks the social processes that shape the variation in net use in practice.^5^ These indicators conceal certain windows in space and time in which socially clustered groups of people at-risk of malaria may be exposed to biting vectors because LLINs are not fully aligned to use in the local sociocultural and environmental conditions. As such, nonuse of LLINs is not randomly distributed within a population, but clustered by certain social characteristics, potentially creating sufficient opportunities for malaria transmission to persist in this pre-elimination setting.

For policy-makers and implementers, the relevance of contextualized findings such as ours is frequently countered with the argument that the minimal increase in LLIN use expected by taking “user preferences” into account is not worth the investment that countries have to make to accommodate those preferences.^43^ Narrowly conceived “user preferences” (e.g., color and shape of nets) alone may indeed not impact on LLIN use substantially in nationally aggregated data, and this logic holds true in settings that are still focused on controlling malaria and where further upscaling of LLINs can substantially reduce transmission. Existing malaria indicators may be appropriate for rapidly assessing the rollout and uptake of LLINs in malaria control settings, but they should not be uncritically extended to address important knowledge gaps in the new malaria elimination era.

In elimination settings, with increasing heterogeneity, where persisting transmission clusters in specific populations and locations despite assumed universal coverage of LLINs, indicators at population level are less useful for assessing critical gaps in coverage. Furthermore, when universal coverage is assumed, persisting malaria transmission is frequently attributed to “residual transmission,” axiomatically defined as all transmission that continues after universal coverage with effective LLINs and IRS has been achieved.^4^ In reality, ongoing transmission may be due to truly “residual” transmission, which in Ratanakiri province is not only caused by early and outdoor biting vectors and outdoor evening and night-time activities^7,44^ but also due to variability in LLIN use partly induced by a lack of appropriateness of the intervention. It remains unclear to what extent “indoor” night-time biting contributes to transmission in Ratanakiri, although our previous research suggests this could still account for almost 50% of the total exposure.^7^ Furthermore, in Ratanakiri, even “early” mosquito biting can often be categorized under “social” nighttime because sleeping times are much earlier than frequently assumed,^7^ as also shown for Vietnam,^45^ and therefore can be addressed with LLINs.

**Figure 1.**
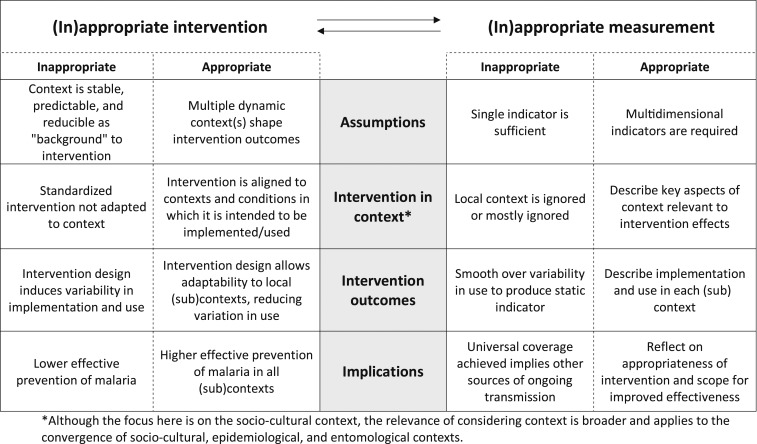
Framework for assessing the appropriateness of the intervention.

In addition to the commonly used criteria of “efficacy” and “effectiveness” when assessing LLINs as a tool for malaria prevention,^46–50^ we have proposed “appropriateness” as a third key concept by which to evaluate LLINs and other global health interventions. We have argued that appropriateness is a concept that applies equally to the measurement of the impact of the intervention as to the intervention itself. Although the concept of appropriateness will require substantial development to be fully realized, we propose a heuristic tool (Figure 1) to aid researchers and program implementers to reflect on how the (in)appropriateness of interventions relates to the (in)appropriateness of indicators. For example, indicators that assume stable behaviors and consistency in LLIN use cannot address variability in LLIN use, in itself partly induced by a lack of appropriateness of the distributed LLINs in the local context. And yet, without recognizing or measuring this variability, programmers lack an “evidence base” to guide optimizing the appropriateness of LLIN interventions in different contexts. More crucially, optimizing the appropriateness of LLIN interventions could meaningfully reduce malaria incidence, including in settings where universal coverage is assumed to have been achieved or nearly achieved. As of yet, other control measures such as (spatial) repellents or insecticide-treated hammocks have not proven their efficacy or cost-effectiveness in interrupting transmission in low transmission settings. Trialing novel technologies should not fully divert attention and resources from the opportunity to reconsider the appropriateness of and increase LLIN use, one of the most effective tools for reducing malaria throughout the malaria-endemic world.

The inappropriateness of measurements used for the evaluation of an intervention cycles back to mischaracterizations of the appropriateness of the intervention, as evident in guidance issued by the WHO for addressing what is implied as suboptimal (and decontextualized) LLIN use.

Where there is evidence that LLINs are not being adequately cared for or used, programmes should design and implement behaviour change communication activities aimed at improving these behaviours (p. 3).^1^

By contrast, optimizing appropriateness could lead to reduced variability in LLIN use, which would in turn render indicators of LLIN use more meaningful. For example, the study population in Ratanakiri required multiple functions from LLINs in addition to protection from biting mosquitoes. Similar to results shown in Peru,^6^ where the authors called for an increased understanding of the “adequacy” of bed nets in the context of implementation, nets created a physical barrier providing users with privacy and prevented dirt from falling onto sleeping spaces from thatched roofs. Also shown in Senegal and Tanzania,^51,52^ nets were mainly valued for their ability to protect from general insect nuisance, including insects smaller than local species of *Anopheles*. By directly engaging with the fact that the most vulnerable households frequently require or adapt household items to have multiple functions, and not dismissing untreated market net use or no net use as misinformed or incorrect, LLINs could be developed to fulfill these additional requirements to increase effective use.^6^ Moreover, the LLINs distributed in Ratanakiri were not thought to be of the quality their specifications claim: in local conditions, these LLIN are reported to deteriorate quickly and are therefore perceived to be inadequate. This lack of appropriateness for local social and environmental conditions has been shown to limit LLIN use in other malaria-endemic settings.^5–7,9,17^

## CONCLUSION

Failure to measure and account for variability in LLIN use leads researchers and programmers to underestimate the continuing potential of LLINs for reducing and interrupting malaria transmission, including the opportunity to improve LLIN appropriateness as a path toward increasing effective use. The importance of these arguments should be recognized in consideration of the shift in research priorities toward interventions for targeting residual transmission, as well as the justification of mass drug administration programs and other elimination interventions, all of which are intended for implementation after malaria control programs assume that universal LLIN coverage has been achieved.
